# Ebstein's Anomaly, Left Ventricular Noncompaction, and Sudden Cardiac Death

**DOI:** 10.1155/2015/854236

**Published:** 2015-07-09

**Authors:** Michael McGee, Luke Warner, Nicholas Collins

**Affiliations:** Cardiovascular Unit, John Hunter Hospital, Newcastle, NSW 2305, Australia

## Abstract

Ebstein's anomaly is a congenital disorder characterized by apical displacement of the septal leaflet of the tricuspid valve. Ebstein's anomaly may be seen in association with other cardiac conditions, including patent foramen ovale, atrial septal defect, and left ventricular noncompaction (LVNC). LVNC is characterized by increased trabeculation within the left ventricular apex. Echocardiography is often used to diagnose LVNC; however, magnetic resonance (MR) imaging offers superior characterization of the myocardium. We report a case of sudden cardiac death in a patient with Ebstein's anomaly with unrecognized LVNC noted on post mortem examination with screening documenting the presence of LVNC in one of the patient's twin sons.

## 1. Introduction

Ebstein's anomaly is an uncommon congenital cardiac disorder characterized by apical displacement of the septal leaflet of the tricuspid valve. While Ebstein's anomaly may occur in isolation, it may also be associated with other cardiac conditions. While the presence of an interatrial communication is seen in more than 70% of patients, other associations include left ventricular noncompaction (LVNC) [1–3]. LVNC is characterized by failure of the usual myocardial development with the condition notable for deep trabeculations, typically within the left ventricular apex. LVNC may be associated with progressive left ventricular dysfunction, development of arrhythmia, systemic embolization, and sudden death. While echocardiography may demonstrate the typical pattern of LVNC, magnetic resonance (MR) imaging offers improved definition [4]. LVNC in those patients with Ebstein's anomaly has been associated with mutations in the sarcomere gene MYH7 [5, 6].

## 2. Case Presentation

We report a case of sudden cardiac death in a patient with Ebstein's anomaly with unrecognized LVNC noted on post mortem examination; subsequent family screening documented the presence of LVNC in one of the patient's twin sons. This case demonstrates the important role of MR imaging in patients with Ebstein's anomaly to document the presence of LVNC, highlights difficulties in stratifying risk for sudden cardiac death in patients with complex congenital heart disease, and demonstrates the potential utility of genetic testing.

A 45-year-old male with a background of known Ebstein's anomaly died suddenly while at work. Ebstein's anomaly had been diagnosed during early childhood, with significant apical displacement of the tricuspid valve leaflet noted with associated tricuspid valve incompetence. The right atrium was markedly dilated with atrialisation of the right ventricle. The patient maintained a satisfactory exercise capacity until the age of 27 years. At that stage, the patient noted a degree of effort intolerance in the context of severe tricuspid valve regurgitation and intermittent complete AV block. There was no history of syncope or features of tachyarrhythmia. The patient then underwent tricuspid valve repair and permanent pacemaker implantation. The patient had an excellent surgical result with improved effort tolerance and mild to moderate residual tricuspid regurgitation. Over the following 14 years, the patient progressed appropriately with preserved exercise capacity, absence of symptoms of arrhythmia, and no clinical features of right heart failure. Echocardiography consistently demonstrated normal left ventricular size and systolic function. There was persisting right atrial and ventricular enlargement with mild to moderate tricuspid valve incompetence. At the time of review prior to death, the patient remained clinically well with no history of syncope or presyncope. Similarly, there was no history of palpitations. The previously inserted pacemaker had been explanted due to long term maintenance of sinus rhythm.

**Figure 1:**
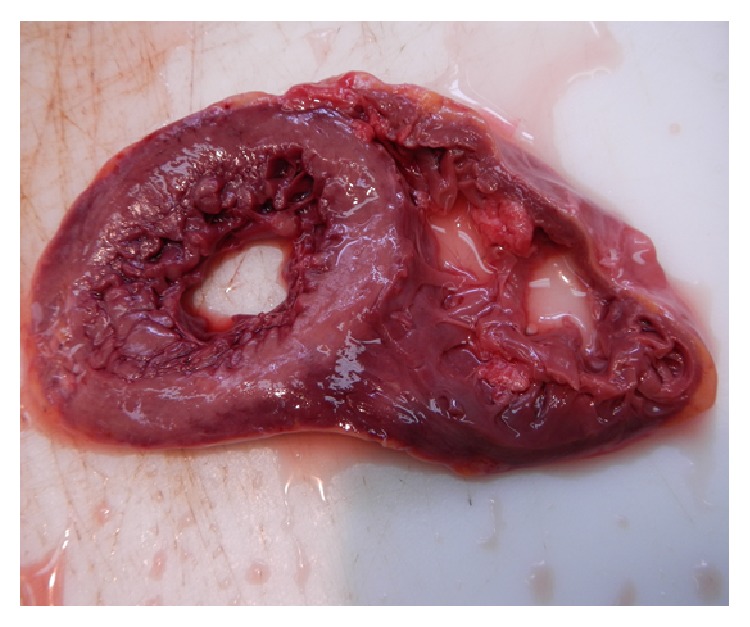
Cross section examination of the apex of the left ventricle demonstrating marked trabeculation within left ventricular cavity.

Post mortem examination demonstrated evidence of previous tricuspid valve surgery with marked right atrial dilatation. The right ventricle was thin walled and dilated. The coronary arteries were normal. The left ventricle was mildly dilated with prominent trabeculation at the apex and posterolateral left ventricular wall segments suggestive of left ventricular noncompaction (LVNC) with accompanying fibrosis noted (Figure 1). No other significant abnormalities were noted at autopsy with the cause of death attributed to malignant cardiac arrhythmia complicating underlying complex cardiac disease.

**Figure 2:**
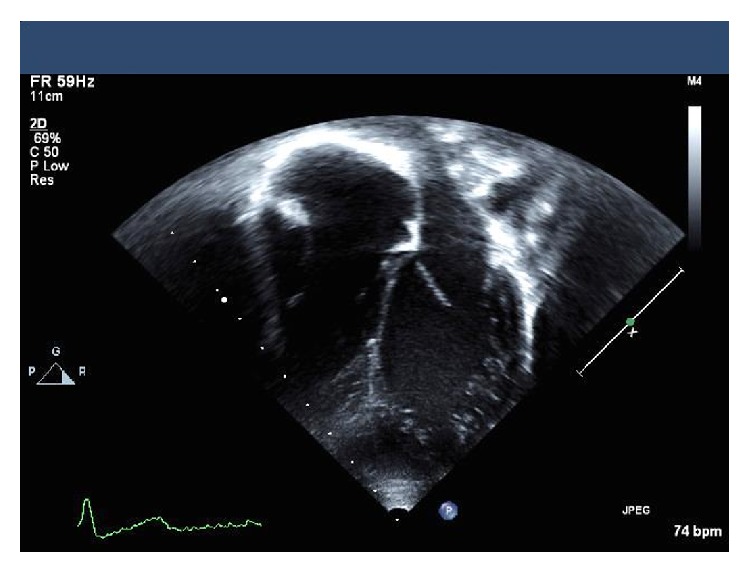
Echocardiography obtained from the apical 4-chamber view demonstrating apical trabeculation consistent with left ventricular noncompaction.

In view of the previously undocumented presence of LVNC, the patient's twin sons underwent echocardiographic screening with one child demonstrating features of LVNC (Figure 2).

## 3. Discussion

This case outlines the importance of recognizing known disease associations in patients with complex congenital heart disease (CHD) and highlights current limitations in risk stratification for sudden cardiac death in CHD, given the patients preserved left ventricular function. Furthermore, given the limitations of echocardiography in documenting LVNC, there may be a role for routine screening of patients with Ebstein's anomaly with MR imaging to exclude LVNC. Finally, given the variable expression of genetic abnormalities associated with Ebstein's anomaly and LVNC, genetic screening may similarly be important in identifying subtle cardiac disease in relatives of patients with both Ebstein's anomaly and LVNC.

LVNC is an uncommon disorder characterized by failure of usual myocardial development with the process of trabecular compaction incomplete. Improvements in imaging techniques have permitted increased recognition of LVNC and allowed for refined diagnostic criteria, utilizing both echocardiography and cardiac MR imaging. The spectrum of disease complicating LVNC is broad, ranging from asymptomatic to severe left ventricular dysfunction with concomitant risks of arrhythmia, systemic embolization, and sudden cardiac death. Predictors of an adverse outcome include earlier age at presentation, impaired functional class, history of ventricular arrhythmia, left ventricular dilatation, and abnormal tissue Doppler parameters [4]. LVNC may occur in isolation or in association with underlying congenital heart disease. In particular, an association with Ebstein's anomaly is recognized [1, 3] with an association with mutations in sarcomere gene MYH7 implicated [5, 6].

The diagnostic criteria for LVNC have undergone evolution with the advent of MR imaging and improved 2D echocardiography imaging quality and are based on the extent and depth of trabeculation within the left ventricle. MR imaging provides superior myocardial characterization and endocardial definition compared to echocardiography and is a more sensitive imaging modality in detecting LVNC. Current guidelines do not advocate routine MR imaging in patients with Ebstein's anomaly; however, in the setting of a family history of sudden death or in cases where there are concerns regarding left ventricular size, appearance, or function, MR imaging may provide valuable additional information. Given the risk of arrhythmia seen in this case, despite preserved ventricular function, there may be a role for routine screening to exclude LVNC. In this case, the patient has undergone serial echocardiography, with no significant left ventricular abnormalities noted. This reflects the limitations of echocardiography in identifying subtle changes in left ventricular myocardial morphology and highlights the important role of MR imaging in diagnosis.

The association between Ebstein's anomaly and LVNC may be related to mutations in the sarcomere gene MYH7. The combination of Ebstein's anomaly and LVNC should prompt formal genetic assessment as well as screening in relatives. As documented in this case, and in previous series, LVNC can manifest without abnormalities of the tricuspid valve.

## 4. Conclusions

This case highlights the difficulties seen in complex congenital heart disease with regard to issues such as risk stratification for sudden cardiac death, the role of advanced imaging modalities such as MR imaging, genetic testing, and screening of family members. The association between Ebstein's anomaly and LVNC is well established with specific genes implicated, noting incomplete penetrance and variable phenotype. Routine MR imaging of patients with Ebstein's anomaly, and potentially their relatives, may be of value in detecting subtle features of LVNC, which in turn has implications in terms of prognosis.
